# Der israelbezogene Antisemitismus und die Gratwanderung einer nicht-antisemitischen ‚Israelkritik‘ – Versuch einer demokratietheoretischen Mediation

**DOI:** 10.1007/s41682-022-00117-0

**Published:** 2022-05-24

**Authors:** Oliver Hidalgo

**Affiliations:** Institut für Politikwissenschaft, Universität Regensburg, Regensburg, Deutschland

**Keywords:** Antisemitismus, Apartheid, BDS, Demokratie, Israel, Westjordanland, Antisemitism, Apartheid, BDS, Democracy, Israel, West Bank

## Abstract

Die wichtige Debatte um den israelbezogenen Antisemitismus ist aktuell in eine Sackgasse geraten. Um zur Versachlichung der wissenschaftlich-öffentlichen Diskussion beizutragen, schlägt der vorliegende Artikel eine Differenzierung von subjektiv bewussten und unbewussten antisemitischen Ressentiments vor und bettet diese Unterscheidung in ein demokratietheoretisches Argument ein. Vor dem Hintergrund eines Demokratiebegriffs, der die komplexe Koexistenz von Einheit und Pluralität in demokratischen Gesellschaften ebenso impliziert wie das grundsätzliche Spannungsverhältnis zwischen Religion und Volksherrschaft, wird es dabei nicht nur möglich, den Staat Israel in nicht-antisemitischer Weise als jüdisches Kollektiv zu reflektieren, sondern auch einschlägige Aporien im Nahostkonflikt zu veranschaulichen. Auf dieser Basis erfolgt eine ausgewogene Auseinandersetzung mit dem von Kritiker:innen lancierten Apartheidvorwurfs gegenüber der israelischen Regierung sowie eine Einschätzung der palästinensischen Boykottbewegung BDS.

## Abstract (en)

The important debate on Israel-centered antisemitism has currently reached an impasse. In order to contribute to the objectification of the academic and public discussion, this article proposes a differentiation between subjectively conscious and unconscious anti-semitic resentments and reformulates this distinction in terms of democratic theory. Against the background of a concept of democracy including the complex coexistence of unity and plurality in democratic societies as well as the fundamental tension between religion and popular sovereignty, it becomes available to reflect the state of Israel as a Jewish collective in a non-antisemitic way, while illustrating relevant aporias in the Middle East conflict at the same time. On this basis, a well-balanced discussion of the apartheid accusation against the Israeli government is as possible as a plausible assessment of the Palestinian boycott movement BDS.

## Einführende Bemerkungen

Der Begriff des ‚neuen‘ Antisemitismus trägt in erster Linie dem Phänomen Rechnung, dass sich judenfeindliche Einstellungen und Handlungen *nach* der Schoah zumindest in demokratischen Gesellschaften unter der Bedingung ihrer offiziellen Ächtung vollziehen. Häufig wird jener ‚neue‘ Antisemitismus als „sekundär“ bezeichnet, da er entlang einer Täter-Opfer-Umkehr den Jüdinnen und Juden (Mit‑)Schuld am Holocaust gibt, um dadurch nicht nur die begangenen Verbrechen, sondern auch mögliche Schuldgefühle und Verantwortungen zu relativieren. Quasi per definitionem scheint sich der ‚sekundäre‘ Antisemitismus zwar jenseits traditionell judenfeindlicher, rechtsextremistischer Milieus abzuspielen, die von der gesamtgesellschaftlichen Verurteilung des Judenhasses erst gar nicht durchdrungen sind. Indes korrespondiert bereits jede Relativierung des Genozids an den Juden gewollt oder ungewollt mit einer Aufwertung der implizit oder explizit vorgebrachten ‚Erklärungen‘, die zu einer wenigstens subjektiven Rechtfertigung der Morde und Gewalttaten herangezogen werden (können). Insofern sind ‚primärer‘ und ‚sekundärer‘ Antisemitismus generell nicht voneinander zu trennen.^1^

Doch noch aus einem weiteren Grund ist die Trennschärfe, die der Begriff des ‚neuen‘ Antisemitismus für sich reklamieren kann, eher gering zu taxieren. Dieser Grund bezieht sich auf den zentralen Adressanten, gegen den sich die Ressentiments des ‚neuen‘ Antisemitismus bevorzugt richten und auf den judeophobe Stereotype, wie sie z. T. seit dem Mittelalter bekannt sind, heute zunehmend projiziert werden: den Staat Israel.^2^ Das ‚Neue‘, dass Israel als Konsequenz der sogenannten Zweiten (oder Al-Aqsa‑)Intifada seit spätestens Herbst 2000 weltweit zum primären Gegenstand und Bezugsrahmen antisemitischer Angriffe geworden ist (Botsch 2019, S. 22), mischt sich demnach mit dem ‚Alten‘, wonach Antisemit:innen ihre traditionellen Vorurteile gegenüber Jüdinnen und Juden anhand der Politik Israels gegenüber der palästinensischen Bevölkerung bestätigt sehen wollen. Umgekehrt bestehen jedoch auch viele, die sich selbst keinesfalls dem antisemitischen Lager zuordnen würden, darauf, dass Israel für seine Behandlung der Palästinenser bzw. den nach wie vor oft ungesicherten Rechtsstatus nichtjüdischer Bevölkerungsgruppen kritisiert werden müsse, ohne dass solche Kritik in die Nähe antisemitischer Positionen gerückt werden dürfe.

In den vergangenen Jahren ist die hieran anknüpfende öffentliche Debatte in eine bisweilen heftig ausgetragene Kontroverse umgeschlagen. Wie oft dabei auf der einen Seite behauptet wurde, der Begriff des ‚neuen‘ Antisemitismus sei geeignet, Israel gegenüber legitimer Kritik an seiner Politik zu immunisieren, während die andere Seite eben darin schon wieder einen (sekundären) antisemitischen Vorbehalt vermutete, ist mittlerweile kaum noch zu überblicken.^3^ Vordergründig drehte sich der Streit in jedem Fall um die Frage, inwieweit solche Kritik an der Politik Israels ausreichend differenziert oder zu pauschal geübt wird. Als neue Form des Antisemitismus wurde es deswegen beschrieben, wenn eine „Übertragung der Kritik an der Politik Israels auf alle Juden“ (Heyder et al. 2005, S. 148 f.) stattfinde oder wenn eine „antisemitisch grundierte Israelfeindschaft“ das Land „Israel [als] kollektiver Jude“ auffasse (Rensmann 2006, S. 33, 345). Auch die „Arbeitsdefinition von Antisemitismus“ der *International Holocaust Remembrance Alliance* (IHRA) aus dem Jahr 2016 nimmt diese Perspektive auf, indem sie auf die Möglichkeit verweist, dass der „Staat Israel, der dabei als jüdisches Kollektiv verstanden wird“,^4^ Ziel von antisemitischen Attacken sein könne. Bei näherem Hinsehen scheint jedoch gerade die Auffassung Israels als „jüdisches Kollektiv“ schwerlich als abstraktes Kriterium für eine Unterscheidung zwischen (legitimer) Kritik an Israel und einer Spielart des ‚neuen‘ Antisemitismus zu taugen. Zum einen liegt dies daran, dass die Artikulation von Kritik an den kollektiv verbindlichen Entscheidungen einer Regierung wie der israelischen nahezu gezwungenermaßen im Modus der Pauschalierung und ohne die parallele Berücksichtigung vorhandener oppositioneller Meinungen erfolgt.^5^ Deswegen ist es im alltäglichen Sprachgebrauch üblich, die Politik der Regierung eines Landes mit den Handlungen der dort lebenden Bevölkerung gleichzusetzen (z. B. „die Amerikaner“, „die Deutschen“, „die Franzosen“ etc.), ohne dass daran zwingend eine pejorative Bewertung dieser Handlungen ablesbar wäre. Das in der IHRA-Definition zuletzt genannte Beispiel einer aktuellen Erscheinungsform des Antisemitismus – „[das] kollektive Verantwortlichmachen von Jüdinnen und Juden für Handlungen des Staates Israel“ – ist in seiner Formulierung somit wenigstens missverständlich und widerspricht zudem tendenziell einer Einschränkung, die von der IHRA selbst betont wird: „Allerdings kann Kritik an Israel, die mit der an anderen Ländern vergleichbar ist, nicht als antisemitisch betrachtet werden.“^6^

Zum anderen aber ist gerade im Fall Israels eine besondere Form der semantischen Kollektivierung festzustellen, die beileibe nicht nur eine Außenansicht suggeriert, sondern die zum zentralen Selbstverständnis des Staates Israel (oder zumindest der Mehrheit der jüdischen Israelis) zählt. Die Rede ist hier vom gleichermaßen säkularen wie politisch-theologischen Fundament, das Israel als dem ‚Nationalstaat der Juden‘ zugrunde liegt, wie es – in verschärfter Form – im 2018 verabschiedeten israelischen Nationalstaatsgesetz zum Ausdruck kommt, welches Israel explizit als „nationale Heimstätte des jüdischen Volkes“ tituliert. Im Staat Israel folglich *kein *jüdisches Kollektiv zu sehen, ist trotz des dort ansässigen arabischen Bevölkerungsteils spätestens seitdem kaum noch möglich.^7^

Aus den bis dato getätigten Überlegungen ergibt sich folgerichtig der Bedarf, den israelbezogenen Antisemitismus, dessen Existenz und Voranschreiten an dieser Stelle keinesfalls bestritten wird, in alternativer Manier zu charakterisieren als in seiner (bloßen) Assoziation mit einem ‚jüdischen Kollektiv‘. Letzteres avanciert in der im weiteren Verlauf des vorliegenden Aufsatzes zu entwickelnden Argumentationslinie vielmehr im Gegenteil zu einem Kriterium, das gerade eine *nicht-antisemitische Kritik* an der Politik des Staates Israel anzuzeigen imstande ist. Eben dies geschieht – wie noch zu zeigen ist – unter der Voraussetzung, dass dieses ‚jüdische Kollektiv‘ Israel als demokratisch organisiert respektiert wird und die einschlägigen Kritikpunkte mithin die demokratische Legitimität dessen, was kritisiert wird, nicht oder aber nur entlang von intersubjektiv nachvollziehbaren Gründen in Abrede stellt (Abschn. 4). Um die Stichhaltigkeit dieses Vorschlags demonstrieren zu können, ist es allerdings nötig, zuvor nochmals kursorisch die Sackgasse zu verdeutlichen, in die sich die bisherige Debatte um den israelbezogenen Antisemitismus manövriert hat. Aus dieser Sackgasse will das hier vorgestellte Argument bestenfalls hinausführen (Abschn. 2). Zur Vorbereitung jener ‚demokratietheoretischen Mediation‘, die geleistet werden soll, führt Abschn. 3 als ein Art Scharnier die Unterscheidung zwischen ‚unbewusstem‘ und ‚bewusstem‘ Antisemitismus ein. Vor diesem Hintergrund sollte es schließlich möglich sein, die jeweiligen Kriterien zu konturieren, wann sich der Diskurs um den israelbezogenen Antisemitismus innerhalb oder außerhalb des demokratischen Referenzrahmens abspielt.

## Indizien einer verfahrenen Debatte

Nach üblicher Lesart manifestiert sich der ‚neue‘ Antisemitismus heute primär in Bezug auf den Nahostkonflikt und erhebt mithin Israel zu seinem primären Gegenstand (Botsch 2019, S. 22 f.). Umgekehrt weisen ‚Israelkritiker‘ die gegen sie erhobenen Antisemitismusvorwürfe meist sehr vehement zurück.^8^ Schwierigkeiten der Bewertung ergeben sich offenbar aus den nahezu unausweichlichen Überschneidungen. Was etwa über eine „sachlich gerechtfertigte Kritik an Israel hinausgeh[t]“ (Rabinovici und Sznaider 2019, S. 10 f.), lässt sich ebenso wenig intersubjektiv verbindlich als Unterscheidungskriterium zum Antisemitismus angeben wie die – an sich richtige – Feststellung der *International Holocaust Remembrance Alliance* (IHRA), wonach auch der „Staat Israel, der dabei als jüdisches Kollektiv verstanden wird, Ziel solcher [antisemitischen] Angriffe sein [kann].“ Entsprechend belässt es die einschlägig formulierte Definition der IHRA bewusst bei dem Konjunktiv, dass Kritik an Israel vor dem Hintergrund antisemitischer Vorbehalte erfolgen *könne*, dies im Umkehrschluss aber keineswegs tun *müsse*. Nicht einmal die bekannten Kategorien des sog. „3-D-Tests“ – Delegitimierung, Dämonisierung und Doppelstandards (Sharansky 2004) – sind bei näherem Hinsehen geeignet, zwischen einer ‚legitimen‘ Kritik an Israel und dem ‚neuen‘ Antisemitismus unmissverständlich zu differenzieren: So muss etwa eine Kritik an der israelischen Siedlungspolitik, die den Staat Israel in seinem (aktuellen, von der Regierung Netanjahu^9^ verkörperten) territorialen Selbstverständnis attackiert, die jüdischen Siedler in ihrem Auftreten gegenüber den Palästinensern als ‚rücksichtslos‘ auffasst und Israel ohne simultane Beanstandung der Geopolitik anderer Länder tadelt, keineswegs notwendig einer antisemitischen Einstellung entspringen – und das, obwohl die 3‑D-Kriterien damit zumindest nach subjektiver Lesart erfüllt sein könnten.^10^ Jede konkret am Staat Israel geübte Kritik ist demnach allenfalls im Einzelfall und schwerlich anhand allgemeingültiger Kriterien auf eventuell vorhandene antisemitische Züge hin zu beurteilen. Plausibel sind lediglich negative Maßstäbe: So wäre eine an Israel vorgebrachte Kritik nicht allein deshalb von jedem antisemitischen Verdacht freizusprechen, wenn es selbst Jüdinnen und Juden sind, die sie äußern, oder weil es sich ggf. um Kritiker:innen handelt, die sich ihres Antisemitismus nicht bewusst sind.^11^ Im Gegenzug aber darf gerade bei Kritiken an der israelischen Politik, deren Urheber selbst jüdisch und/oder ihrer aufrichtigen Wahrnehmung nach keine Antisemit:innen sind, ein solcher Vorwurf nicht vorschnell erhoben werden.

Genau letzteres scheint jedoch allem Anschein nach im Rahmen der öffentlichen Debatte in den letzten Jahren zunehmend zu passieren. Drei markante Beispiele eines offenbar hoffnungslos verfahrenen Diskurses sollen an dieser Stelle zur Illustration genügen.

- In einem offenen Brief an Bundeskanzlerin Angela Merkel vom 24. Juli 2020,12 drückten über 60 deutsche und israelische Wissenschaftler:innen und Intellektuelle – darunter Katajun Amirpur, Aleida und Jan Assmann, Wolfgang Benz, Micha Brumlik, Naomi Chazan, Gideon Freudenthal, Amos Goldberg, Sten Nadolny, Fania Oz-Salzberger, Klaus Staeck, Moshe Zimmermann oder Moshe Zuckermann^13^ – ihre Sorge darüber aus, dass sich im Kontext der „völkerrechtswidrigen Besatzungs- und Siedlungspolitik“ Israels mittlerweile ein „inflationäre[r], sachlich unbegründete[r] und gesetzlich unfundierte[r] Gebrauch des Antisemitismus-Begriffs“ etabliert habe, „der auf die Unterdrückung legitimer Kritik an der israelischen Regierungspolitik“ abziele. Als Beispiel für das „menschenverachtende Ausmaß“, welches jene [angebliche, AdV] Instrumentalisierung des Antisemitismusvorwurfs angenommen habe, weisen die Unterzeichner:innen des Briefes auf das Buch *Der neu-deutsche Antisemit* (2018) von Arye Sharuz Shalicar hin, in welchem der Historiker und Publizist Reiner Bernstein als „Antisemit geschmäht“ werde, obwohl er sich lediglich „für eine gerechte und gewaltfreie Lösung des Israel-Palästina Konflikts ein[setze]“. Als Abteilungsleiter im Büro des (damaligen) israelischen Premierministers verkörpere Shalicar somit in paradigmatischer Manier die Strategie Netanjahus, „jegliche Kritik der völkerrechtswidrigen Besatzungs- und Siedlungspolitik als antiisraelisch und antisemitisch zu brandmarken“. Dies sei umso mehr zu beanstanden, weil die israelische Regierung damit eine ähnliche „politische Haltung“ an den Tag lege wie einst der Mörder Jitzchak Rabins, der durch seine Gewalttat den seinerzeit erfolgsversprechenden Friedensprozess zwischen Israel und Palästina zum Erliegen brachte, und weil zugleich von „realen antisemitischen Gesinnungen und Ausschreitungen ab[gelenkt]“ werde. In seiner Replik auf diese Vorwürfe verfasste der Zentralrat der Juden in Deutschland einige Tage später am 29. Juli 2020 seinerseits einen offenen Brief^14^ an die Bundeskanzlerin, in dem er die entgegengesetzte Sorge über den aktuellen „politischen und wissenschaftlichen Umgang mit Antisemitismus“ äußerte, welcher oftmals mit „persönliche[r] Diffamierung“ einhergehe und den leider alltäglichen Antisemitismus in Deutschland verharmlose. „Sehr häufig“ werde dabei „Antisemitismus als Kritik am israelischen Staat kaschiert“, entgegen den Intentionen der Arbeitsdefinition der IHRA, die Kritiker zu Unrecht als Angriff auf die freie Meinungsäußerung verurteilten. Insofern unterstütze und begrüße der Zentralrat es ausdrücklich, wenn die Antisemitismus-Beauftragten der Bundes- und Landesregierungen kritisch aufzeigen, „wo der jüdische Staat dafür herhalten muss, um Judenfeindlichkeit zu transportieren“.^15^ Dass die im Zuge der IHRA-Definition entbrannte wissenschaftliche Debatte stattdessen davon ablenke, „wo es wirklich Antisemitismus zu bekämpfen gilt“, könne der Zentralrat ebenso wenig feststellen wie dass dadurch „Kritiker der israelischen Regierung mundtot“ gemacht würden. Und wenn sich in diesem Zusammenhang „Wissenschaftler gegenseitig mit heftigen Angriffen überziehen“, dann stehe dies nicht in der Verantwortung derer, die vor dem Hintergrund der IHRA-Definition allgemein auf antisemitische Tendenzen aufmerksam machen.
  Worauf *beide* offenen Briefe indes allenfalls implizit eingehen, ist, mit welcher Begründung Shalicar Bernstein überhaupt Antisemitismus vorwarf. In dieser Hinsicht formulierte der offene Brief vom 24. Juli 2020 lediglich die spekulative Vermutung, dass „in Deutschland eine Stimmung der Brandmarkung, Einschüchterung und Angst“ vorherrsche, der es ebenso zuzuschreiben sei, dass „das Berliner Kammergericht Bernsteins Klage gegen seine Verleumdung [als Antisemiten] zurückgewiesen“ habe. Ein Kommentar eines Unterzeichners des offenen Briefes an Merkel, Micha Brumlik (2020), präzisierte diese Vermutung später dahingehend, dass es sich bei jener „Stimmung“ um die gleiche (übersensible) Atmosphäre handle, die nicht nur „Shalicars Angriffe auf Reiner Bernstein ermutigt habe“, sondern die auch am 17. Mai 2019 zu dem Bundestagsbeschluss führte, der „mit großer Mehrheit […] die gewaltfreie (!) palästinensische BDS-Bewegung [als] antisemitisch“ deklarierte. Indem nun allerdings der Zentralrat der Juden in seiner Reaktion auf den realen Hintergrund der antiisraelischen Kampagne BDS (Boycott, Divestment and Sanctions) gar nicht einging, war sein Schreiben von vornherein ungeeignet, einer womöglich berechtigten Sorge über ggf. stattfindende Instrumentalisierungen des Antisemitismusvorwurfs überzeugend zu begegnen. Umgekehrt aber kam die Schärfe im offenen Brief der israelkritischen Intellektuellen offenkundig weniger durch die Verteidigung Bernsteins als durch die polemische Suggestion zustande, die israelische Regierung würde wenigstens indirekt die Geisteshaltung repräsentieren, die 1994 zum Mord an Rabin motivierte, und damit mittlerweile auch die deutsche Regierung infiltrieren.
  Mit anderen Worten, das eigentliche Problem der ganzen Debatte – die BDS-Bewegung (Hänel 2020) – wurde von beiden Seiten explizit umschifft, um im Gegenzug jedoch jeweils implizit eine mögliche Gegenposition zu diskreditieren. Die Einstufung eines bekennenden BDS-Verteidigers (wie Bernstein) als Antisemiten^16^ erscheint dadurch den einen als gefährlicher Missbrauch eines überaus gravierenden Vorwurfs, während die anderen per se die Option förmlich ausklammern, dass sich ein Verteidiger von BDS eventuell zu Recht und (inter)subjektiv überzeugend gegenüber dem Verdikt des Antisemitismus verwehrt. Eine solche wechselseitig implizierte Zuspitzung der Argumentation bewirkt fast notgedrungen eine Form der Polarisierung,^17^ in der von vornherein jede Verständigung ausgeschlossen ist, selbst zwischen denen, die im Grunde gleichermaßen den Kampf gegen den Antisemitismus unterstützen wie offen für legitime Kritik an der israelischen Regierungspolitik sind.
- Die innerisraelische Kontroverse über die Siedlungspolitik der Regierung Netanjahu wurde in ihrer Vehemenz vor allem davon belegt, dass die linke und arabische Opposition verstärkt den Begriff ‚Apartheid‘ verwendete, um die aus ihrer Sicht erfolgende Diskriminierung der palästinensischen Bevölkerung anzuprangern.^18^ Die einschlägige Debatte spitzte sich nochmals zu, nachdem der 2019 geschlossene Koalitionsvertrag zwischen den Regierungsparteien Ministerpräsident Netanjahu ermächtigte, Teile des besetzten Westjordanlands dem israelischen Staat einzuverleiben. Von einer solchen Annexion betroffen sollten nach Aussagen von Netanjahu im vorherigen Wahlkampf nicht nur die bisher bestehenden jüdischen Siedlungen, sondern auch das Jordantal sein, das heißt bis zu einem Drittel des gesamten besetzten Gebiets (Croitoru 2020).
  Dass die Entrechtung der nach dem Krieg von 1948 auf israelischem Gebiet verbliebenen Palästinenser:innen, die bis 1966 einer repressiven Militärverwaltung unterstellt waren und auch danach von Polizei und Geheimdienst streng kontrolliert blieben, Parallelen mit dem ehemaligen Apartheidregime in Südafrika aufweist, ist politisch zwar umstritten, mit Blick auf die Anti-Apartheidkonvention der Vereinten Nationen 1973 jedoch gerade für die heutigen Besatzungsgebiete sowie die weitgehende Segregation der Palästinenser:innen von der jüdischen Mehrheitsgesellschaft schwer zu entkräften. Denn auch wenn die meisten Befürworter:innen der israelischen Besatzungs- und Siedlungspolitik darauf pochen, dass sich dahinter keinerlei rassistische Motive, sondern einzig sicherheitspolitische Erwägungen verbergen, ist nicht von der Hand zu weisen, dass gerade in den besetzten Gebieten wesentliche der in Artikel 2 der UN-Konvention festgelegten Kriterien erfüllt sind. Dazu zählt, dass im Westjordanland die Palästinenser:innen anders als die jüdischen Siedler:innen bis heute der Militärgerichtsbarkeit unterstellt sind, ihre Bewegungsfreiheit durch ein rigides System von Passierscheinen, Checkpoints und Verboten massiv eingeschränkt ist, die Infrastrukturen inklusive der Straßen, Strom- und Wasserversorgung getrennt bleiben, Festnahmen z. T. ohne Haftbefehl erfolgen oder auch willkürlich anmutende Enteignungen von Besitz stattfinden. All dies lässt sich im weiteren Sinne durchaus als „vorsätzliche Schaffung von Bedingungen“ interpretieren, „welche die volle Entwicklung der unterdrückten Gruppe verhindern“ (Croitoru 2020).
  Gleichzeitig ist kaum zu übersehen, wie der semantische Vergleich mit dem rassistischen Apartheidsystem in Südafrika die Komplexität des Israel-Palästina-Konflikts ignoriert und zu einer einseitigen Schuldzuweisung bzw. einer simplen Täter-Opfer-Dichotomie einlädt, die nach Lage der Dinge nahezu unausweichlich Missverständnisse, Verzerrungen sowie unangemessene Pauschalierungen produziert (Hebel 2021). Dass in einem solch polemischen Diskussionsklima damals wie heute der Apartheidvorwurf der einen vom Antisemitismusvorwurf der anderen Seite gekontert wird, vermag kaum zu überraschen. Dies hat sich insbesondere in der heftigen Kontroverse um Achille Mbembe gezeigt, der in seinem Vorwort zum Buch *Apartheid Israel *(Soske und Jacobs 2015) die Meinung äußerte, die Besetzung Palästinas sei „the biggest moral scandal of our times, one of the most dehumanizing ordeals of the century“ (Mbembe 2015, S. viii). In seinem eigenen Buch *Politik der Feindschaft* bekräftigt Mbembe (2017, S. 85), die israelische Siedlungspolitik „erinnere in mancherlei Hinsicht“ an die Apartheid in Südafrika, um sodann in einer anderen Passage „das Apartheidregime in Südafrika“ und „die Vernichtung der europäischen Juden“ als „zwei emblematische Manifestationen“ des gleichen kolonialen Grundprinzips zu diagnostizieren, das er als „Trennungswahn“ bezeichnet (ebd., S. 89). Indem Mbembe den Begriff der Apartheid hier allerdings als „Metapher“ (ebd., S. 85) verwendet und den Holocaust durch den Einschub „– in einer ganz anderen Größenordnung und in einem anderen Kontext“ (ebd., S. 89) explizit vom rassistischen Regime in Südafrika abgrenzt, demonstriert er damit offenkundig seine Absicht, die genannten Phänomene gerade *nicht gleichsetzen* zu wollen. Nichtsdestoweniger warf ihm der Antisemitismusbeauftragte der Bundesregierung, Felix Klein, vor, in seinen Werken „durch die Relativierung des Holocaust auf[zu]fallen“ sowie „den Staat Israel mit dem Apartheidsystem Südafrikas gleich[zusetzen]“, „was einem bekannten antisemitischen Muster“ entspreche. Auch „das Existenzrecht Israels“ sei von Mbembe „in Frage gestellt“ worden,^19^ ohne dass Klein dies näher belegen würde.
  Wie sich die Diskursmacht des zur Beschreibung des Verhältnisses zwischen Israelis und Palästinensern lancierten Apartheidbegriffs in prekärer Manier zu verselbständigen droht, wenn vorhandene Indizien zu undifferenziert zu seiner Bestätigung (oder auch Entkräftung) herangezogen werden,^20^ hat sich ebenso im Kontext der COVID-19-Pandemie verdeutlicht. So griff beispielsweise die *Frankfurter Rundschau* die Metapher einer völkerrechtswidrigen Apartheidpolitik auf, um die Impfpolitik Israels seit Ende 2020 zu kritisieren (Borchert 2021):^21^ Millionen Palästinenser:innen seien von der Regierung Netanjahu im Stich gelassen worden, obwohl nach *Human Rights Watch* aus der Vierten Genfer Konvention die Pflicht Israels resultiere, die medizinische Versorgung nicht nur der jüdischen Siedler:innen, sondern auch der Palästinenser:innen in den besetzen Gebieten sicherzustellen.^22^ Demgegenüber insistierten die israelischen Behörden zunächst darauf, dass das Gesundheitswesen gemäß der Osloer Verträge der Verantwortung der palästinensischen Autonomiebehörde unterstellt sei, bevor das Kabinett in Jerusalem aufgrund der wachsenden Kritik an seiner Position Ende Januar 2021 beschloss, 5000 Dosen des Impfstoffes *Moderna* an die Palästinenser:innen weiterzureichen (Stahnke 2021). Vergessen werden sollte dabei nicht, dass palästinensische Pendler:innen schon zuvor Teil des israelischen Impfprogramms waren und die Autonomiebehörde zunächst von sich aus deklariert hatte, ausreichend Impfstoff besorgen zu wollen.^23^
  Erneut aber ging der Streit um Apartheid und Antisemitismus in der Hauptsache am grundlegenden Problem vorbei, nämlich dass für eine erfolgreiche Bekämpfung der Pandemie im Westjordanland die Kooperation zwischen Autonomiebehörde und israelischer Regierung unerlässlich war und ist. Die verfahrene Situation mit gegenseitigen Schuldzuweisungen resultierte insofern in erster Linie daraus, dass die eingangs beschriebenen Annexionsankündigungen der Netanjahu-Regierung die Beziehungen zwischen Israelis und Palästinensern schon im Vorfeld der Pandemie extrem belasteten und jede Form der Zusammenarbeit in der Krise massiv erschwerten. Ohne daher an dieser Stelle stattgefundene Versäumnisse auf beiden Seiten entschuldigen zu wollen, hat die COVID-19-Krise hier wie in so vielen anderen Fällen weniger als Ursache, denn als verstärkender Katalysator für zuvor ungelöste Probleme gewirkt. Die Kontraproduktivität der Apartheid vs. Antisemitismus-Debatte hätte insofern kaum eindrucksvoller demonstriert werden können.
- In der Neuauflage des Bandes *Neuer Antisemitismus? Fortsetzung einer globalen Debatte* (2019, Hrsg. Christian Heilbronn et al.) sind Doron Rabinovici und Natan Sznaider (2019, S. 11 f.) in ihrer aktualisierten Einleitung offensichtlich sehr um eine ausgewogene Darstellung der einschlägigen wissenschaftlichen und öffentlichen Diskussion bemüht. Deswegen versuchen sie, die soeben in a) und b) skizzierte, seit Längerem verfahrene Kontroverse um die Unterscheidbarkeit von legitimer Kritik an der israelischen Regierungspolitik und antisemitischen Ressentiments als Produkt einer wechselseitigen „Rhetorik des Verdachts“ zu entlarven, mit der „alle Beteiligten der Debatte arbeiten“ würden. Dabei gründe der Antisemitismusvorwurf in diesem Zusammenhang „auf der Vermutung, dass das Gesagte nicht das Gemeinte ist – dass Kritik an Israel nur ein Vorwand ist, um antisemitische Ideen oder Gefühle zu artikulieren, bewusst oder auch unbewusst“. Demgegenüber argwöhne die andere Seite, „der Antisemitismusvorwurf diene nur dem Interesse Israels, legitime Kritik zum Schweigen zu bringen“. Beide Seiten, so Rabinovici und Sznaider, lägen „mit ihren Verdächtigungen [zuweilen wohl] nicht ganz daneben“. Dies würde folgerichtig zur *doppelten* Vorsicht mahnen, sowohl antisemitische Vorbehalte hinter kritischen Stellungnahmen gegenüber Israel wahrzunehmen als auch achtsam gegenüber einem eventuell vorschnell erhobenen, unbegründeten Antisemitismusvorwurf zu sein, der – ebenfalls bewusst oder unbewusst – einer möglichen Immunisierungsstrategie gegenüber Kritik etwa an der israelischen Siedlungspolitik Vorschub leisten könnte.
  Umso kontraproduktiver mutet es in dieser Hinsicht an, wenn Jan Süselbeck (2019) in seiner Besprechung des von Rabinovici und Sznaider mitherausgegebenen Bandes bereits den bloßen *Versuch* einer solchen Ausgewogenheit als „enttäuschend[e] Unentschiedenheit des Urteils“ bezeichnet, die angesichts der „dramatisch gewachsenen weltweiten Gefährdung von Jüdinnen und Juden“ vollkommen unangebracht sei. Zu dieser einseitigen Stellungnahme kann Süselbeck gelangen, weil er zwar die antisemitischen Hintergründe zahlreicher ‚Israelkritiken‘ registriert, es aber als „Phantasma“^24^ und „nachweislich falsche Behauptung“ abtut, dass legitime Kritik an Israel mithilfe des Antisemitismusvorwurfs wenigstens in bestimmten Fällen tabuisiert wird. Dazu beruft er sich einmal mehr auf den 3‑D-Test, mit dem verbindlich anzugeben sei, wann eine „sogenannte Israel-Kritik den Tatbestand der offenen Delegitimierung, Dämonisierung und der Benutzung doppelter Standards“ erfülle und somit keine „rationale politische Einschätzung, sondern Antisemitismus“ vorliege.^25^ Zum anderen rekurriert Süselbeck auf Monica Schwarz-Friesels Studie *Antisemitismus 2.0*,^26^ die trotz weitreichenden Online-Recherchen „noch nicht einmal eine einzige seriöse Gleichsetzung von Israel-Kritik mit Antisemitismus gefunden habe“. Damit sei „belegt“, dass es sich beim „Topos des Israel-Kritik-Tabus“ um eine reine „Fiktion handelt“. Das Ganze sei eine „Schein-Debatte“, um sich als „Opfer eines ‚Meinungsdiktats‘ zu gerieren und den eigenen Antisemitismus zu kaschieren bzw. als ‚Kritik‘ umzudeuten und damit akzeptabel“ zu machen.
  Wie die in a) und b) nachgezeichneten Beispiele demonstrieren, ist die fragliche Kontroverse indes deutlich komplexer als sich bloß um die Frage zu drehen, ob Israel-Kritik mit Antisemitismus ‚gleichgesetzt‘ wird oder nicht. Stattdessen werden durch Süselbecks Position wenigstens implizit auch diejenigen, die sich in jener „Schein-Debatte“ lediglich zu Wort melden, weil sie die *Möglichkeit* einer Instrumentalisierung des Antisemitismusvorwurfs einräumen, dem Verdacht ausgesetzt, freiwillig oder unfreiwillig Handlanger:innen antisemitischer Einstellungen zu sein. Eine ‚seriöse‘ Auseinandersetzung mit einer Auffassung wie bspw. derjenigen von Micha Brumlik (2019), der den aktuellen Antisemitismusdiskurs mit einer neuen Form des McCarthyismus vergleicht, wäre auf Basis der Position Süselbecks nicht nur unnötig, sondern sogar gefährlich und problematisch für den Kampf gegen antisemitische Vorurteile.^27^ Damit gießt er jedoch Wasser auf die Mühlen all derer, die etwa die Ansicht Brumliks teilen und vor der Instrumentalisierung des Antisemitismusvorwurfs im Zusammenhang mit Kritik an der israelischen Regierungspolitik warnen. Aus dieser Perspektive wäre eine Auffassung wie die Süselbecks, die ein Vermittlungsangebot à la Rabinovici und Sznaider ausschlägt und zur neuerlichen Verhärtung der Fronten beiträgt, sogar dezidiert als *Teil *einer solchen Immunisierungsstrategie zu lesen, deren Phänomen Süselbeck selbst wie gesehen als „Phantasma“ deutet. Völlig ausgeblendet aber droht dadurch zu werden, dass es allen eben genannten Beteiligten dieser verkorksten Debatte nachweislich darum geht, Antisemitismus keinen Raum zu geben. Der Streit dreht sich hier lediglich darum, ob es dafür notwendig ist, eine Pauschalverurteilung derjenigen abzulehnen, die BDS verteidigen bzw. die Parallelen zwischen Israel zu einem Apartheidregime betonen, oder aber umgekehrt die Nähe zwischen solchen Argumentationslogiken und antisemitischen Vorbehalten zu unterstreichen.

Als übergreifendes Kardinalproblem des wie gesehen extrem polarisierten Diskurses um den israelbezogenen Antisemitismus lässt es sich daher ausmachen, dass es bislang an den nötigen Differenzierungsleistungen fehlt, um die Sackgasse, in die sich die einschlägige Diskussion manövriert hat, zu verlassen. Solche Differenzierungen aber werden momentan nicht allein dadurch erschwert, dass der Weg zur Relativierung bei keiner Form der Differenzierung weit ist, sondern vor allem auch dadurch, dass alle, die sich in die fragliche Debatte einmischen, mit ihren Stellungnahmen die Vorwürfe der Gegenseite performativ bestätigen, insbesondere dann, wenn es ihnen explizit darum geht, einen neutralen Standpunkt einzunehmen. Denn im Kontext (der Bekämpfung) des Antisemitismus ist Neutralität schlicht nicht hinreichend.^28^ Gleichwohl gilt es, nach geeigneten Unterscheidungen Ausschau zu halten, um eine konstruktivere wissenschaftlich-öffentliche Debatte zu arrangieren als in der Vergangenheit. Der folgende Abschnitt wird in dieser Hinsicht den bereits bei Rabinovici und Sznaider angeklungenen Vorschlag aufgreifen, die Frage des israelbezogenen Antisemitismus nicht länger entlang der bisher üblichen Attribute eines angeblich ‚neuen‘ gegenüber einem ‚alten‘ Antisemitismus zu behandeln, sondern besser mit Hilfe der Unterscheidung von subjektiv bewussten oder unbewussten Vorurteilen. Nicht um letztere zu bagatellisieren, wohl aber, um die Diskursmacht der Radikalen auf beiden Seiten zu reduzieren und die Verständigung zwischen denen zu erleichtern, die unter den aktuellen Voraussetzungen den Kampf gegen den Antisemitismus als gemeinsames Anliegen miteinander teilen.

## Bewusster vs. unbewusster Antisemitismus

Die in diesem Abschnitt vorgenommene Unterscheidung zwischen subjektiv bewussten und unbewussten antisemitischen Ressentiments ist sich über ihre eigenen Unzulänglichkeiten und die dadurch per se begrenzte Reichweite des Arguments vollauf im Klaren. So ermöglicht die vorgeschlagene Differenzierung keineswegs eine jederzeit eindeutige, dichotome Aufteilung vorhandener Positionen, sondern sind Grauzonen, Überlappungen oder auch Oszillationen zwischen beiden Polen vorprogrammiert, gerade wenn das „Unbewusste“ diesbezüglich mit latenten, nicht ausreichend reflektierten Vorurteilen einhergeht. Nicht in Abrede wird außerdem gestellt, dass es keinen „harmlosen Antisemitismus“ gibt (Klein 2021), sondern dass auch subjektiv unbewusste antisemitische Stereotypen und Vorurteile grundsätzlich nicht zu tolerieren sind. Vor allem aber bildet die Unterscheidung zwischen „bewusstem“ und „unbewusstem“ Antisemitismus gewiss kein Patentrezept, wie die notwendige Versachlichung im gegenwärtigen politischen und wissenschaftlichen Antisemitismusdiskurs zu bewerkstelligen sein könnte, sondern dürfte auf eigene Weise zu fundiertem Widerspruch provozieren.

Die Applikation der entsprechenden Semantik besitzt gleichwohl solch eminente Vorzüge, dass die genannten Schwächen dadurch überwogen werden sollten. Dies ist im Folgenden kurz zu erläutern Zunächst hat bereits die Rekapitulation des verfahrenen Diskurses um den israelbezogenen Antisemitismus gezeigt, dass die Diskussionspartner:innen jeweils von der Existenz unbewusster Vorbehalte auf der Gegenseite überzeugt sind. Es ist mithin gar nicht strittig, ob es so etwas wie unbewusste antisemitische Ressentiments gibt, sondern allenfalls, ob sich diese heute bevorzugt in der Formulierung von Kritiken an der israelischen Regierungspolitik niederschlagen bzw. inwieweit ein Antisemitismusvorwurf ggf. unbeabsichtigt als Immunisierungsstrategie für Israels Politik genutzt wird. Dies macht das Phänomen unbewusster oder zumindest nicht ausreichend reflektierter antisemitischer Vorbehalte zu einem adäquaten Anknüpfungspunkt für eine konstruktiv gelagerte Debatte, nicht zuletzt, weil diese in womöglich unbegründeten Antisemitismusvorwürfen (die dadurch ungewollt der Tabuisierung berechtigter Kritik an der israelischen Politik dienen) ein plausibles Pendant finden.

Hinzu kommt, dass die beidseitig feststellbaren Pauschalierungen, die im Kontext der Debatte um den israelbezogenen Antisemitismus eine Verhärtung der Fronten provoziert haben, allem Anschein nach vielen der beteiligten Akteure oft tatsächlich nur am Rande evident sind. Für die Reihe an ‚Israelkritikern‘ die sich selbst in keiner Weise als Antisemiten sehen, sollte es diesbezüglich ein durchaus besorgniserregendes Detail sein, dass sich der Begriff ‚Israelkritik‘ im öffentlichen Sprachgebrauch etabliert hat, ohne dass sich eine vergleichbare Semantik zur Verurteilung der Politik anderer Staaten herausgebildet hätte. Dass es Wörter wie ‚Russlandkritik‘, ‚Türkeikritik‘, ‚Chinakritik‘ oder auch ‚Amerikakritik‘ laut Duden nicht gibt, schürt die Vermutung, dass bei der Bewertung israelischer (Macht‑)Politik tatsächlich exzeptionelle Standards und Ansprüche einfließen, die für die anderen genannten Länder nicht gelten – geschweige denn für europäische Nachbarn wie Frankreich, Italien oder Großbritannien. Ein wenigstens schleichender Antisemitismus – vergleichbar mit dem ‚Antiamerikanismus‘, der offenkundig über eine ‚legitime‘ Kritik an den USA hinausgeht – ist hier schwerlich zu übersehen und durchaus von der Frage zu trennen, inwieweit die israelische Regierungspolitik etwa aus humanitärer Sicht zur Kritik herausfordert. Desgleichen haftet einer transnationalen Bewegung wie BDS, die zu „Boykott, Desinvestitionen und Sanktionen“ gegen den „Staat Israel“ aufruft, ohne hinreichend zwischen israelischer Regierung und Gesellschaft zu unterscheiden, die Situation der arabischen Israelis differenziert zu betrachten und vor allem das Existenzrechts Israels unmissverständlich anzuerkennen, unweigerlich ein wenigstens latent antisemitischer Charakterzug an.^29^ Unter diesen Voraussetzungen eine aktive Unterstützung von BDS subjektiv nur als „Israelkritik“ zu deklarieren und von jeglichen antisemitischen Vorbehalten freizusprechen, mag daher zur Selbstberuhigung beitragen, ist jedoch intersubjektiv kaum überzeugend. Im Gegenzug scheint allerdings ebenso wenig zu bestreiten, dass mangels plausibler Alternativen auch oder sogar primär nicht-antisemitische Einstellungen zur Unterstützung von BDS motivieren können und damit bekundete Sympathien für die Anliegen der Palästinenser:innen im Nahostkonflikt nicht automatisch mit Antisemitismus gleichzusetzen sind. Nachvollziehbare Skepsis gegenüber einer einschlägigen ‚Israelkritik‘ mitsamt ihren nahezu unvermeidlich delegitimierenden, pejorativen Implikationen oder auch das grundsätzliche Verständnis für das Sicherheitsbedürfnis Israels dürfen hier infolgedessen zu keinem Generalverdacht gegenüber der einen sowie zur vorauseilenden Entschuldigung der anderen Konfliktpartei führen. Mit anderen Worten, die angebrachte Sensibilität gegenüber einem Antisemitismus, der bei zahlreichen ‚Israelkritiken‘ subjektiv unreflektiert mitschwingt, darf umgekehrt nicht blindmachen für die Aspekte der israelischen Regierungspolitik, deren Rechtfertigung die Grenzen der Legitimität und Verhältnismäßigkeit sprengen würde. So ist es nicht zufriedenstellend, die von internationalen wie israelischen Organisationen dokumentierten Menschenrechtsverletzungen im Westjordanland^30^ sowie Kritik an den Annexionsplänen der Regierung Netanjahu^31^ als reine antisemitische Propaganda abzutun,^32^ auch wenn es viele Antisemit:innen gibt, die nur allzu bereitwillig Verfehlungen Israels zur Bestätigung ihrer Ressentiments heranziehen und zugleich Menschenrechtsverletzungen von palästinensischer Seite oder auch von anderen Staaten ignorieren.^33^ Das eine Unrecht wird durch ein anderes nicht aufgewogen, diese im Grunde banale Erkenntnis sucht man im Kontext der Kontroverse um den israelbezogenen Antisemitismus bisweilen vergeblich.

Folglich genügt es auch nicht, festzustellen, dass die „Form“ des israelbezogenen Antisemitismus heute „dominant“ wird und sich darin „alle Antisemiten [treffen]“ (Schwarz-Friesel 2020b): Weil – bis auf wenige Ausnahmen^34^ – Antisemit:innen in der Regel Kritiker:innen Israels sind, jedoch nicht alle Kritiker Israels Antisemiten, wirkt es je nach Standpunkt plausibel, die Gefahr des israelbezogenen Antisemitismus zu *über*- oder zu *unter*schätzen – und die Gegenseite zugleich zu brüskieren. Weder die Israelisierung des Antisemitismus noch die Antisemitisierung von Kritik an Israels sind deswegen zu verharmlosen. In einem emotional höchst aufgeregten Diskurs, bei dem jede Art der Versachlichung schwerfällt,^35^ braucht es stattdessen umso mehr Differenzierungen, die die Polarisierung nicht unnötig weiter anheizen, sondern potenziell zur Entschärfung der Kontroverse angetan sind.

Eine solche Entschärfung ist von der Unterscheidung respektive Akzeptanz von subjektiv unbewussten und bewussten Ressentiments auf beiden Seiten bis zu einem gewissen Grad zu erwarten, weil der oben konstatierten reziproken „Rhetorik des Verdachts“ in der Debatte über den ‚neuen‘ Antisemitismus dadurch etwas Signifikantes entgegengesetzt wird. Anstatt den Eindruck zu erwecken, Befürchtungen seien jeweils nur in einer Richtung angebracht, wird sowohl konzediert, dass „Kritik an Israel“ eventuell „nur ein Vorwand ist, um antisemitische Ideen oder Gefühle zu artikulieren“, als auch, dass sich „der Antisemitismusvorwurf“ im Gegenzug instrumentalisieren lässt, um dem „Interesse Israels“ zu dienen und „legitime Kritik zum Schweigen zu bringen“ (Rabinovici und Sznaider 2019, S. 12). Anstatt also zu behaupten, nur das eine könne passieren, das andere aber nicht, um damit erst recht den Argwohn zu wecken, zwischen ‚Gesagtem‘ und ‚Gemeintem‘ bestehe unter Umständen eine folgenschwere Diskrepanz, impliziert die wechselseitige Akzeptanz subjektiv unbewusster Ressentiments nicht nur die möglicherweise unreflektierte Position des Diskussionspartners, sondern die gleiche Option *ebenso bei einem selbst*. Dieselbe Einsicht fordert, die Spirale aus (womöglich ja auch falschen) Verdächtigungen und Unterstellungen nicht endlos fortzusetzen, sondern wenigstens die Eventualität einzuräumen, *der/die andere könnte mit seinen/ihren Vermutungen Recht haben*. Im Gegenzug verlangt eben dies von allen beteiligten Gesprächspartner:innen, die jeweils eigene, zur Disposition stehende Wahrnehmung mit konkreten, nachvollziehbaren Argumenten zu belegen, sei es, um die Vorwürfe der anderen Seite zu entkräften, sei es, um bestehende eigene Vorbehalte zu konkretisieren. Das heißt, in jedem Einzelfall gilt es unter der Voraussetzung einer womöglich subjektiv unbewussten Affinität zum Antisemitismus bzw. einer ebenfalls unbewussten Strategie der Immunisierung israelischer Politik im besonderen Maße empirisch nachvollziehbar zu demonstrieren, wann und von wem eine ‚legitime‘ Kritik an Israel (subjektiv) tatsächlich zu Unrecht als Antisemitismus verunglimpft wurde oder wann umgekehrt eine (subjektiv) als Kritik an Israel formulierte Verurteilung jene judenfeindlichen Stereotypen, Ressentiments und Pauschalierungen aufweist, die sie als antisemitisches Vorurteil decodierbar macht. Beides geschieht in der Debatte bislang viel zu selten, sodass die ‚Rhetorik des Verdachts‘ und der Unterstellung meist nicht überwunden, sondern perpetuiert wird. Solange indes gar keine intersubjektiv nachprüfbaren und somit diskutierbaren Belege für Antisemitismus respektive eine Instrumentalisierung des Antisemitismusvorwurfs erbracht werden, die Ebene des Konjunktivs also zu keiner Zeit verlassen wird, wäre im Grunde genommen von vornherein große Zurückhaltung im Urteil angesagt – bei allen Beteiligten. Eine solche Zurückhaltung liegt ebenso dem potenziellen Zugeständnis eigener unbewusster Ressentiments in die eine oder andere Richtung zugrunde.

Zweifelsohne am wichtigsten an der Unterscheidung von bewusstem und unbewusstem Antisemitismus jedoch ist, dass Menschen und Gruppen, in deren individuellem oder kollektivem Bewusstsein sich unbemerkt „antisemitische Stereotype eingegraben“ haben (Schwarz-Friesel 2019b, S. 388), nicht ohne Weiteres mit denjenigen in ein und denselben Topf geworfen werden dürfen, die antisemitische Vorurteile vorsätzlich lancieren bzw. bewusst codieren, um dem Straftatbestand der Volksverhetzung zu entgehen. In dieser Hinsicht ist zu betonen, dass nicht etwa eine solche Unterscheidung den Antisemitismus und die in seinem Gefolge begangenen Verbrechen zu relativieren droht, sondern exakt die Verweigerung einer solchen Distinktion. Wenn kein Unterschied mehr besteht zwischen solchen Einstellungen, die bspw. Juden pauschal für eine subjektiv verfehlte israelische Regierungspolitik verurteilen, ohne den dahinterstehenden latenten Antisemitismus zu bemerken, und solchen, die den Genozid an Millionen von Juden gutheißen, dann wird damit nicht möglichen Anfängen gewehrt, sondern auch denjenigen ein mörderischer Judenhass attestiert, die von einem solchen de facto weit entfernt sind. Vor allem aber ist – insbesondere im Rahmen der Demokratie und der demokratischen Diskussions- und Entscheidungsverfahren – mit denjenigen ein ‚konstruktiver‘, tendenziell ‚toleranterer‘ Umgang ebenso möglich wie geboten, die subjektiv *keine* Antisemit:innen sein *wollen*, auch wenn sie in ihren Einstellungen objektiv bzw. intersubjektiv antisemitische Züge aufweisen sollten.

Diese Auffassung ist keineswegs damit gleichzusetzen, Antisemitismus allein in Form von (rechts-)extremen „Gesinnungen und Ausschreitungen“ als „real“ sowie als „tatsächlich[e]“ Gefährdung jüdischen Lebens in Deutschland und Europa anzuerkennen.^36^ Stattdessen ist nicht zu ignorieren, dass sekundärer Antisemitismus, der Jüdinnen und Juden dezidiert eine (Mit‑)Schuld an ihrer Diskriminierung und Verfolgung in Geschichte und Gegenwart gibt, allenfalls in einem gesellschaftlichen Umfeld attraktiv und (subjektiv) ‚überzeugend‘ sein kann, in dem primärer Antisemitismus nach wie vor akut oder latent wirkungsmächtig ist. Nicht um die Bagatellisierung eines unreflektierten ‚Alltagsantisemitismus‘ geht es, sondern um eine möglichst klare Trennlinie, wie antisemitischen Ressentiments erfolgreich zu begegnen ist: mit geduldiger Aufklärung, Empathie und sachlicher Diskussion oder aber mit aktivem Widerstand, politischer Kompromisslosigkeit und strafrechtlicher Ahndung.^37^ Auch strukturelle Komponenten des Antisemitismus sind in diesem Zusammenhang nicht zu vernachlässigen: So ist im Vergleich mit anderen Arten der Diskriminierung als Besonderheit des Antisemitismus zu berücksichtigen, dass Jüdinnen und Juden für gewöhnlich nicht dafür gehasst werden, was sie *nicht* sind oder haben, sondern eben dafür, was sie haben oder sind (Horvilleur 2020, S. 14). Als vielerorts beobachtete Profiteure des jeweiligen gesellschaftlichen Systems ziehen sie daher häufig Neid und Eifersucht auf sich und werden so v. a. in Krisensituationen leicht als Sündenböcke identifiziert – bis hin zum bekannten Paradox des „Antisemitismus ohne Juden“ (Lendvai 1972), bei dem strukturähnliche Feindbilder erfahrungsgemäß leicht auf Jüdinnen und Juden projiziert werden. Um es indes zumindest zu erschweren, dass solche (eingebildeten) Feindbilder in offenen, gewalttätigen Judenhass umschlagen, ist es vonnöten, subjektiv unbewusste antisemitische Ressentiments soweit es eben geht von bloßen Vorwänden für manifeste antisemitische Einstellungen zu differenzieren. Andernfalls wäre es nicht nur keine Option, durch Bewusstmachen solcher unbewusster Vorurteile zu deren Überwindung und dadurch zu einer Eindämmung des Antisemitismus beizutragen; sondern schlimmstenfalls würden dadurch diejenigen, die allenfalls latente antisemitische Ressentiments besitzen, geradewegs in die Arme derjenigen getrieben, die offenen Antisemitismus predigen.^38^

## Eine demokratische Mediation des Problems? Zwischen Einheit und Konflikt

Die im vorherigen Abschnitt als dringend notwendig identifizierte Unterscheidung von bewusstem und unbewusstem Antisemitismus respektive einer bewussten oder unbewussten Immunisierung israelischer Politik mag auf den ersten Blick von rein (sozial-)psychologischem Interesse sein und kaum das Potenzial beherbergen, zur politischen Entspannung der Fronten im Kontext des israelbezogenen Antisemitismus beizusteuern. Schließlich bleiben die beidseitigen Verdächtigungen ja bestehen bzw. wird dadurch sogar implizit bekräftigt, dass sich zwischen ‚Gesagtem‘ und ‚Gemeintem‘ eine Kluft auftut. Auch und gerade entlang der Differenzierung von unbewussten und bewussten Vorurteilen auf beiden Seiten wird mithin eine klare, intersubjektiv verbindliche Trennung von legitimer Kritik an der Politik Israels und antisemitischen Vorurteilen nicht wahrscheinlicher, im Gegenteil: Sobald unbewusste Einstellungen als Teil des Problems akzeptiert werden, scheint eine einseitige Polemik in der einschlägigen politischen Debatte kaum zu vermeiden, indem sich die Betroffenen im Zweifelsfall gegen die (subjektiv als solche empfundene) ‚Unterstellung‘ unreflektierter Ressentiments oftmals nicht weniger wehren dürften als gegen den Vorwurf manifester Vorurteile – zumindest solange, wie das subjektiv ‚Unbewusste‘, womöglich aber gar nicht ‚Gewollte‘ anhält.

Gleichwohl scheint eine konstruktive Wende des Diskurses auf Basis der vorgeschlagenen Unterscheidung erreichbar, sofern ihre *demokratietheoretischen* Implikationen ins Blickfeld geraten. Um das betroffene Problem zunächst zu illustrieren, sei auf eine Äußerung des ehemaligen Bundesministers Norbert Blüm verwiesen, der 2002 gegenüber dem damaligen israelischen Botschafter Schimon Stein die *Operation Schutzschild* als „hemmungslosen Vernichtungskrieg“ bezeichnete.^39^ Die unter diesem Namen bekannt gewordene Militäroperation wurde von der israelischen Armee im Westjordanland zur Verhinderung weiterer palästinensischer Attentate während der zweiten Intifada durchgeführt. Das Kardinalproblem von Blüms antisemitisch wenigstens eingefärbter Äußerung war es dabei *nicht*, dass sie in ihrer Kritik an den Maßnahmen der Regierung Ariel Scharon die zuvor stattgefundenen palästinensischen Terrorakte ignorierte oder wenigstens nicht als Entschuldigung gelten ließ (Schubert 2019, S. 146). Schließlich muss auch der Anti-Terrorkampf einer Demokratie wie Israel rechtsstaatliche Grenzen akzeptieren, sodass die Verhältnismäßigkeit der israelischen Großoffensive auch von vielen anderen Beobachtern bezweifelt wurde. Womit Blüm allerdings offenbar unbewusst^40^ antisemitische Klischees bediente, war die Wortwahl des ‚hemmungslosen Vernichtungskriegs‘, mit der er ohne hinreichende historische Sensibilität sowie in Verkennung der jeweiligen Opferdimensionen eine Art Gleichsetzung zwischen der *Operation Schutzschild* und dem Russland-Feldzug von Nazi-Deutschland im Jahr 1941 suggerierte oder zumindest billigend in Kauf nahm (Joffe 2018). Am Kern der Problematik im Nahostkonflikt zielte Blüm damit vorbei, lässt sich der Staat Israel doch zu keiner Zeit seiner Gründung als eine totalitäre Diktatur auffassen, die auf die Vernichtung anderer Völker aus ist, selbst wenn im Rahmen der Sicherheits- und Siedlungspolitik im Westjordanland auch unschuldige Zivilisten ums Leben kamen und es in Israel wie in anderen Ländern radikale, fanatische, rassistische und muslimfeindliche Stimmen gibt, für welche die (Menschen)Rechte der Palästinenser:innen nicht viel zählen. Als *staatliche Gesamtheit, *als Kollektiv jedoch verkörpert Israel kein Regime, das gegen die palästinensische Bevölkerung einen Vernichtungsfeldzug führt, sondern eine Demokratie, die von einer terroristischen Gruppierung in einem asymmetrischen Konflikt kalkuliert zu Überreaktionen provoziert wird, um ihre Legitimitätsgrundlage in innen- und außenpolitischer Hinsicht zu untergraben, ganz so, wie es bekanntermaßen zur üblichen Kommunikationsstrategie von Terrorist:innen gehört.^41^

Eben diese zentrale Nuance – Israel ist ein demokratischer Staat, der wie andere westliche Demokratien von Terrorist:innen herausgefordert wird und in der Bekämpfung des Terrorismus nicht selten über das Maß der Bedrohung hinausschießt – muss nun auch in der Debatte über den israelbezogenen Antisemitismus als Benchmark herangezogen werden. In dieser Hinsicht wäre es bspw. legitim, die vor allem von den Regierungen Scharon und Netanjahu vorangetriebene israelische Siedlungs- und Besatzungspolitik im Westjordanland oder auch die mangelnde Verhältnismäßigkeit von Militäroffensiven im Anti-Terrorkampf zu kritisieren, ohne dass dies als antisemitisches Vorurteil gebrandmarkt werden dürfte. Der Respekt vor der demokratischen Legitimität der israelischen Regierungen, die diese Politiken zu verantworten hatten und haben, aber verbietet im Gegenzug etwa Vergleiche oder gar Gleichsetzungen mit einem ‚Kampf um Lebensraum‘ oder auch einem ‚Vernichtungskrieg‘, wie ihn einst die Nationalsozialisten betrieben.

Was auf den ersten Blick vielleicht wie eine allzu simple Einsicht, fast eine Binsenweisheit anmutet, die der Komplexität des Problems im Hinblick auf den israelbezogenen Antisemitismus nicht gerecht wird, präsentiert sich bei näherem Hinsehen als eine durchaus plausible formale (nicht inhaltliche) Orientierung(sgrenze): An der Frage des *Umgangs* mit anderen Ansichten sowie insbesondere dem politischen Gegner entscheidet sich letztlich nicht nur die Demokratie, sondern zugleich die Qualität von antisemitischen Äußerungen respektive Antisemitismusvorwürfen. Eine offenbar unbewusste und ungewollte antisemitische Einstellung, wie wir sie vorhin bei Norbert Blüm identifiziert haben, ist als solche deshalb zwar keinesfalls zu verharmlosen, sie lässt sich jedoch *noch* im Rahmen einer demokratischen Diskussion ansiedeln, da dem Urheber von entsprechenden Äußerungen im Zweifelsfall begreiflich zu machen ist,^42^ warum seine Ausdrucksweise antisemitisch imprägniert ist und weshalb eine grundsätzlich legitime Anwaltschaft für palästinensische Opfer bzw. palästinensische Interessen davon eher beeinträchtigt als unterstützt wird. Umgekehrt aber wäre eine nicht-antisemitische Kritik an der israelischen Regierungspolitik im Westjordanland stets (notwendig, wenn auch nicht hinreichend) eine Form, die die demokratischen Vorgänge und Hintergründe, welche einer solchen Politik zugrunde liegen, nicht ausblenden, sondern zum eigentlichen Gegenstand der Kritik machen. Letzteres wäre etwa der Fall, wenn die israelische Regierung dafür gerügt wird, aus machtpolitischem Kalkül Zugeständnisse an radikale Gruppierungen in der Siedlungsfrage zu machen und/oder zu diesem Zweck eventuell sogar dauerhafte Koalitionen einzugehen. Der Rahmen des ‚Unbewussten‘ und damit der traditionell ‚lernfähigen‘ Demokratie^43^ wird indes spätestens dann verlassen, wenn Kritiker:innen das ‚Jüdisch sein‘ als Erklärung für eine subjektiv oder auch intersubjektiv verfehlte Regierungspolitik heranziehen und somit politische (Fehl)Entscheidungen nicht als grundsätzlich kritisierbare Entwicklungen in demokratischen Verfahrensprozessen auffassen, sondern als programmierte Resultate eines unterstellten ‚jüdischen‘ Essenzialismus. In diesem Fall würde ignoriert, dass politische Gegensätze und Konflikte unterschiedlicher Couleur in einer Demokratie niemals aufgehoben, sondern lediglich auf eine bestimmte Weise ausgetragen werden. Mit anderen Worten, dass eine Demokratie wie die israelische kollektiv verbindliche Entscheidungen über festgelegte Verfahren evoziert, die auch die ggf. überstimmte Opposition mitzutragen hat,^44^ darf nicht mit der Suggestion einer Einheit verwechselt werden, die die dahinter stehenden politischen Konflikte gar nicht mehr wahrnimmt bzw. von einem angeblich übergreifenden jüdischen ‚Volkscharakter‘ absorbiert glaubt. Stattdessen ist demokratische ‚Einheit‘ immer nur im Sinne von einschlägigen Theoretiker:innen wie Hans Kelsen,^45^ Hannah Arendt, Claude Lefort, Chantal Mouffe oder Jacques Rancière zu denken, welche die kollektiven Verbindlichkeitsstrukturen der Demokratie mit dem Respekt vor der unauflöslichen Pluralität der Werte und Interessen amalgamiert und somit nicht nur das Gespür für pragmatische Notwendigkeiten, sondern auch für die Aporien jeder konkreten Subjektivierung ‚des‘ Volkes behält.^46^ Das Gleiche gilt für die Formen des Konsenses, der sich im Kontext der Volksherrschaft vor allem auf die friedliche Austragung von inhaltlich unlösbaren Konflikten sowie die Akzeptanz der Existenzberechtigung und demokratischen Legitimität politischer Gegenstimmen beschränkt (vgl. Heller 1971; Mouffe 2007).

Die *demokratisch-pluralistische* Kontroverse, die sich an der israelischen Sicherheitspolitik gegenüber den Palästinenser:innen sowie insbesondere der Siedlungspolitik im Westjordanland sowohl innerisraelisch als auch international entzündet hat, ist insofern performativ als doppeltes (formales) Signum dafür zu lesen, dass Kritik an der einschlägigen Regierungspolitik weder in antisemitische Klischees und Pauschalierungen abdriftet noch durch einen ggf. unberechtigten Antisemitismusvorwurf tabuisiert wird. Dass dieses Signum von den Kontrahent:innen selbst allerdings zum Teil gar nicht mehr als solches wahrgenommen wird, zeigt nicht nur die Verfahrenheit des einschlägigen Diskurses (siehe Abschn. 2), sondern, dass die Kontroverse mittlerweile ein Niveau bzw. eine Dimension eingenommen hat, die ihrerseits den demokratischen Rahmen zu verlassen drohen. Denn dass die einen den anderen hier (manifesten) Antisemitismus unterstellen bzw. umgekehrt eine Immunisierungsstrategie vermuten, belegt, dass beide Gruppen wenigstens dazu tendieren, sich nicht länger als *legitime* politisch-demokratische Gegner zu akzeptieren und sich entsprechend auch an kein *fair play *im Sinne von Herrmann Heller (1971, S. 428) mehr gebunden sehen. Diese Entwicklung ist zum einen losgelöst von der behandelten Thematik als Teil einer allgemein fortschreitenden sozialen Polarisierung in westlichen Demokratien zu sehen.^47^ Zum anderen aber scheint das Thema des israelbezogenen Antisemitismus aktuell eine sehr bedenkliche Eigendynamik zur weiteren Vertiefung der politischen Gräben zu entfalten. Umso wichtiger wäre es, die erst vor Kurzem vollzogene Abwahl von Premierminister Netanjahu als Indiz für die grundsätzliche Intaktheit der israelischen Demokratie zu bewerten. Entsprechend wäre auch die Politik der Regierung Netanjahu in der Rückschau in dem hier vorgeschlagenen Sinne differenziert und vor dem Hintergrund der Vielstimmigkeit der politischen Ansichten in Israel mitsamt ihrer demokratischen Austragungsweise zu bewerten.

Dazu gehört es freilich, auch nochmals die demokratietheoretische Problematik des 2018 verabschiedeten Nationalstaatsgesetzes zu unterstreichen, in dem für zahlreiche Kritiker:innen gerade eine Priorisierung des jüdischen Gemeinschaftscharakters gegenüber demokratisch-rechtsstaatlichen Prinzipen sowie individuellen Grund- und Menschenrechten zum Ausdruck kommt (Lintl 2018).^48^ Zwar lässt sich das Gesetz seinerseits als Resultat eines legitimen demokratischen Prozesses interpretieren, am massiven Widerstand der politischen Opposition, der Zivilgesellschaft und des juristischen Apparats ließ sich jedoch bereits damals ablesen, dass die israelische Demokratie an dieser Stelle Gefahr lief, die vorhin angesprochene notwendige ‚Einheit‘ jenseits aller Parteigrenzen einzubüßen. Dass das israelische Nationalstaatsgesetz das Risiko einer (noch stärkeren) Ausgrenzung der arabisch-palästinensische Minderheit in sich birgt, ist jedenfalls schwerlich von der Hand zu weisen. Zwar wurde die heftig umstrittene Klausel, wonach homogene jüdische Gemeinden auf der Grundlage von Nationalität und/oder Religion zu schaffen sind, am Ende aus dem Gesetzestext gestrichen. Ein anderer, ebenso strittiger Punkt, fand dafür allerdings Eingang, nämlich die de-facto-Nachordnung des Arabischen hinter das Hebräische, ein Passus, der die notwendige demokratische Balance zwischen Einheit und Pluralität^49^ sowie den rechtsstaatlich gebotenen Minderheitenschutz wenigstens der Tendenz nach aufkündigt. Ganz zu schweigen davon, dass das Prinzip der Gleichheit, das bis dato in keinem Grundgesetz Israels festgehalten wurde, auch in diesem Gesetz wieder fehlt (Lintl 2018), nicht zuletzt, um zu verhindern, dass der Oberste Gerichtshof in Israel der arabischen Bevölkerung kollektive Rechte zuspricht.^50^

Dass die Regierungskoalition unter Netanjahu aus rechtskonservativen, ultra-orthodoxen und nationalreligiösen Parteien zunehmend illiberale Positionen vertrat^51^ und nicht zuletzt die jüdische Dominanz im gesamten „Eretz Israel“ (also in Israel und den besetzten palästinensischen Gebieten) beanspruchte, ist insofern eine Tatsache, die sich in erster Linie demokratietheoretisch kritisieren und abseits von antisemitischen Klischees akzentuieren lässt. Zumindest eine „Verengung demokratischer Spielräume“ ist zugleich durch die Verfestigung der Besatzung im Westjordanland sowie die erwähnten Menschenrechtsverletzungen durch das israelische Militär bzw. die Siedler:innen zu konstatieren (Asseburg 2017).

Wie sehr diese problematische Entwicklung vor allem auch innerhalb Israels teilweise zu einem bemerkenswerten Umdenken geführt hat, ist abschließend am Beispiel von Benjamin Pogrund zu zeigen, einem in Südafrika geborenen Israeli, der den Apartheidvorwurf gegenüber Israel lange Zeit eben mit dem Argument abgelehnt hatte, dass diese Semantik der Legitimität und Integrität der israelischen Demokratie in keiner Weise gerecht werde (Pogrund 2005). In einem Interview mit der israelischen *Times* im Juni 2020, das im Kontext der Annexionspläne Netanjahus und Bennetts erfolgte, gab Pogrund allerdings zu, seine Meinung diesbezüglich geändert zu haben: „[At] least it has been a military occupation. Now we are going to put other people under our control and not give them citizenship. That is apartheid. That is an exact mirror of what apartheid was [in South Africa].“^52^ Damit ist nicht ausgemacht, dass der Vorwurf inhaltlich zutrifft, wohl aber, dass er sich unabhängig von der Problematik des israelbezogenen Antisemitismus diskutieren lässt.

## Fazit

Am Ende seiner bahnbrechenden Studie *Der Antisemitismus als Gruppenerscheinung* (1926) prophezeite Carl Bernstein einst, dass die Gründung eines Staates Israel das Problem des Antisemitismus entschärfen würde, weil „ein jüdisches Volk, das geschlossen in seinem eigenen Land lebt“, zwar „den Anfeindungen seiner Nachbarvölker ausgesetzt sein“ wäre, es sich dabei aber um eine „normale Feindschaft von Volk zu Volk“ handeln würde, ohne jenen „einseitige[n], fluchbeladene[n] Haß“ hervorzurufen, „der die Trümmer des gequälten [jüdischen] Volkes durch zwei Jahrtausende und über die ganze bewohnte Erde gejagt hat“ (Bernstein 1926, S. 222 f.). Wie wir heute wissen, hat sich diese Vorhersage nicht bewahrheitet. Stattdessen hat die Gründung des Staates Israel antisemitische Vorbehalte lediglich bis zu einem gewissen Grad neu bzw. anders geschürt als dies in der Vergangenheit der Fall war. Gleichzeitig sind bei jenem ‚neuen‘, israelbezogenen Antisemitismus die alten Ressentiments stets erkennbar.

Die Antwort, warum die Gründung eines jüdischen Staates mit der Herausforderung des Antisemitismus eng verwoben blieb, hat Bernstein indes nur wenige Seiten zuvor selbst gegeben: Hier schreibt er, dass es „gleichgültig“ sei, „wie die Juden sind, was sie tun und lassen“, weshalb es ebenso „zwecklos“ wäre, „wenn sie versuchen, durch ihr Verhalten den Antisemitismus zu beseitigen“ (ebd., S. 220).^53^ Auf die hier behandelte Thematik gemünzt, mündet dies in die Einsicht, dass es heute weder die Wohltaten noch mögliche Verfehlungen der demokratisch legitimierten israelischen Regierung sind, die Antisemitismus provozieren oder eindämmen. Der Referenzpunkt der Demokratie impliziert jedoch – wie evident geworden seine sollte – immerhin die Option, eine kritische Diskussion über israelische Regierungspolitik führen zu können, ohne dass die (mittlerweile verfahrene) Debatte über den israelbezogenen Antisemitismus zwangsläufig tangiert wird.

Der Grat, auf dem in dieser Hinsicht gewandert wird, ist nichtsdestoweniger sehr schmal. Daran ändert auch der hier vorgestellte Versuch, zwischen Antisemitismus und Kritik an der Regierungspolitik Israels entlang des Kriteriums der Demokratie zu differenzieren und dieses Kriterium wiederum entlang der Unterscheidung von subjektiv bewussten und unbewussten Ressentiments zu spiegeln, nur bedingt etwas. Dies liegt zum einen daran, dass ein unbewusster, ungewollter Antisemitismus zwar (demokratie-)theoretisch signifikant und im Vergleich zu einem bewussten, manifesten Antisemitismus sogar der entscheidende Unterschied sein kann, in der aktuellen diskursiven Praxis aber schwer identifizierbar bleibt, seitdem es „nach Ausschwitz“ „um den bekennenden Antisemitismus [ohnehin] still geworden“ ist (Diner 2019, S. 459). Die nach dem Holocaust „hervorgegangene[n] Partikel des Ressentiments“ mögen dabei vom Merkmal ‚bewusst vs. unbewusst‘ de facto allenfalls unzureichend einzufangen sein (ebd.).^54^ Zum anderen aber fließen selbstredend auch in die Bewertungen der israelischen Demokratie antisemitische Vorurteile ein – bewusst oder unbewusst. Israel in dieser Hinsicht vorschnell als ‚Unrechtsstaat‘ zu titulieren, ohne ausreichend zu registrieren, dass sich die israelische Demokratie nicht anders als andere Demokratien schwertut, im Kampf gegen den Terrorismus die Verhältnismäßigkeit zu bewahren, besitzt dabei ebenso eine antisemitische Note wie die Ignoranz dessen, dass sich ultra-orthodoxe, nationalreligiöse Juden mit Demokratie, Pluralität und Menschenrechten auch nicht schwerer tun als radikale Gläubige nichtjüdischer Konfessionen. Abgesehen davon, dass in dem so komplexen Konflikt zwischen Israelis und Palästinensern einseitige Täter-Opfer-Diktionen ohnehin wenig zielführend sind.

Insofern stellt es die grundlegende Ambivalenz der israelischen Staatsgründung dar, dass der Antisemitismus in dieser Form eines jüdischen Kollektivs auf Dauer einen klar identifizierbaren Gegenstand gefunden hat, obwohl dieser aufgrund seiner demokratischen Verfassung eigentlich nicht zum pauschalen Feindbild taugt. Womit sich das eigentliche Kennzeichen des Antisemitismus erneut bestätigt hätte, dass er sich unabhängig vom jüdischen Leben manifestiert.

## Footnotes

1 Damit ist nicht in Abrede gestellt, dass sich der Antisemitismus der Gegenwart durch große Flexibilität und eine wenigstens zum Teil veränderte Trägerschaft auszeichnet (vgl. Laqueur 2008). Waren es bis zur ersten Hälfte des 20. Jahrhunderts vor allem christliche Kirchen, faschistische Bewegungen und konservative Parteien, in denen antisemitische Einstellungen dominierten, konzentriert sich der Antisemitismus unter dem Eindruck des Israel-Palästina-Konflikts heute stärker im Umfeld des Islamismus, des arabischen Nationalismus sowie in linken antizionistischen Bewegungen. Von einer fulminanten Verschiebung kann gleichwohl keine Rede sein, nicht nur, weil der Antisemitismus in rechtsradikalen, rechtskonservativen bzw. auch religiös-fundamentalistischen Milieus nach wie vor sehr präsent ist und seinen „Anteil am Bezug auf den Nahostkonflikt“ nimmt (Botsch 2019, S. 23), sondern, weil der in arabischen und islamisch geprägten Staaten kultivierte Antisemitismus zumindest bis zu einem bestimmten Grad über entsprechende Migrationsbewegungen nach Europa reimportiert wird.

2 Für die nachweisbare Vermischung von Versatzstücken des ‚alten‘ und ‚neuen‘ Antisemitismus, wie sie heute besonders im Internet zu beobachten sind, siehe Schwarz-Friesel (2019a). Dort fließen u. a. die Ergebnisse der Korpusuntersuchung des DFG-Projekts „Antisemitismus im World Wide Web“ (2014–2018) ein. Zu Analogien im Sprachgebrauch zwischen ‚altem‘ und ‚neuem‘ Antisemitismus siehe zudem Schwarz-Friesel und Reinharz (2013).

3 Für den Versuch einer Dokumentation der (Meilensteine der) Debatte im deutschsprachigen Raum siehe nichtsdestotrotz z. B. Schubert (2019) und Wetterau (2020).

4 https://www.holocaustremembrance.com/de/node/196 (16.03.2022).

5 Zu diesem Alleinstellungsmerkmal des politischen Systems, für die Gesamtgesellschaft kollektiv verbindliche Entscheidungen generieren zu können, siehe Luhmann (2002).

6 Die auf Basis der IHRA-Definition wenigstens erschwerte Differenzierung zwischen Antisemitismus einerseits und einer kritischen Haltung gegenüber Israels Politik und Selbstverständnis anderseits hat in jüngerer Zeit zu zwei einschlägigen Klärungsversuchen geführt: der *Jerusalem Declaration on Antisemitism* (siehe dazu den Beitrag von Sigel in dieser Special Section der ZRGP) sowie dem *Nexus-Document* einer Taskforce der *University of Southern California*, die beide im März 2021 veröffentlicht wurden und sich von der IHRA-Begrifflichkeit in teilweise signifikanter Manier abgrenzen. Im Rahmen des vorliegenden Beitrags wird auf diese beiden Dokumente jedoch nicht explizit rekurriert, zum einen, weil sich die zentralen Etappen der hier rekonstruierten Debatte bereits zuvor abgespielt haben, zum anderen, da zum gegenwärtigen Zeitpunkt noch nicht einzuschätzen ist, ob die beiden Dokumente einen vermittelnden oder weiter polarisierenden Charakter besitzen werden.

7 Für die Mehrheit der jüdischen Israelis handelte es sich bei dem mit 62 zu 55 Stimmen (2 Enthaltungen) vor allem auf Betreiben der nationalreligiösen Partei *Jüdisches Heim*, von Teilen des konservativen *Likud* sowie von der säkular-nationalistischen Partei *Israel Beitenu *verabschiedeten Nationalstaatsgesetz ohnehin nur um eine rechtliche Kodifizierung der Tatsache, dass Israel bereits als jüdischer Staat gegründet worden war. Vgl. Lintl (2018). Auch die in Israel geltenden exklusiven Regeln des Staatsbürgerrechts dienen offensichtlich dem demographischen Ziel, die jüdische Mehrheit im Land zu bewahren (Averbukh und Kranz 2016).

8 Für ein breite Palette an Beispielen siehe die unter Anm. 3 genannten Titel.

9 Es ist kaum zu erwarten, dass sich dieses Selbstverständnis durch die seit Juni 2021 amtierende Regierung des nationalreligiösen Premierministers Bennett verändern wird. Stattdessen verlieh Bennett der Forderung einer Annexion größerer Teile des Westjordanlands in der Vergangenheit großen Nachdruck. Siehe Abschn. 2b.

10 Rensmann (2006, S. 345 f.) benennt als viertes Kriterium einer antisemitisch grundierten Israelfeindschaft neben der drei-D-Methode zusätzlich die Verwendung antisemitischer Stereotype im Diskurs um Israel. Genau betrachtet kann es sich dabei allerdings um kein eigenständiges Kriterium zur Unterscheidung von Kritik an Israel und Antisemitismus handeln, weil sich an dieser Stelle Explikat und Explikandum vermischen.

11 Dazu weiterführend Abschn. 3.

12 https://www.tagesspiegel.de/downloads/26044046/1/offener-brief-an-angela-merkel.pdf (16.03.2022).

13 Vor allem Zuckermann (2010) hatte schon zuvor in vehementer Weise versucht, den Antisemitismusvorwurf gegenüber vielen linken und jüdischen Intellektuellen, die die Politik der ‚rechten‘ Regierung Israels gegenüber der palästinensischen Bevölkerung seit Längerem kritisieren, als „Herrschaftsinstrument“ zu entlarven.

14 https://www.zentralratderjuden.de/fileadmin/user_upload/pdfs/Schreiben_an_Bundeskanzlerin_Dr._Angela_Merkel.pdf (16.03.^2022^).

15 In dieser Hinsicht hatte der offene Brief der über 60 Intellektuellen (Anm. 12) eine einseitige und mithin problematische Unterstützung bzw. sogar „Förderung“ der Position Shalicars durch den Antisemitismus-Beauftragten der Bundesregierung Felix Klein suggeriert. Demgegenüber stellt sich der offene Brief des Zentralrats eindeutig auf die Seite Kleins.

16 Hier sollte nicht unter den Tisch fallen, dass die BDS-Bewegung keine hinreichende Differenzierung zwischen dem Staat bzw. der Regierung Israels und der israelischen Gesellschaft vornimmt und dass führende Vertreter von BDS das Existenzrecht Israels zum Teil offen bestreiten. Dazu auch die Abschn. 3 und 4.

17 Jene Polarisierung wurde nicht zuletzt davon geschürt und angeheizt, dass sich der Konflikt um die israelische Siedlungspolitik innen- wie außenpolitisch bevorzugt zwischen ihren rechtskonservativen Befürworter:innen und den linkspolitischen Gegner:innen des früheren Premierministers Netanjahu abspielte. Vgl. Posener (2020).

18 Siehe dazu die entsprechenden Misstrauensanträge in der Knesset, in denen die linke Meretz-Partei sowie die arabisch dominierte Vereinte Liste im Juni 2020 vor einem Apartheidsystem warnten. Schon 2015 hatte der Faschismusforscher Zeev Sternhell in der linksliberalen Tageszeitung Ha’aretz die israelische Besatzungspolitik als Angriff auf die Gleichberechtigung der Palästinenser:innen sowie als Entwicklung kritisiert, die zu einer „kolonialen Realität“ und „offenen Apartheid“ zu führen droht.

19 Zit. nach Häntzschel (2020). In einem Interview mit der ZEIT erwähnt Klein zusätzlich Mbembes Aufsatz *The Society of Enmity* (Mbembe 2016) als Beleg für eine Schrift, die „alle Merkmale des israelbezogenen Antisemitismus [trage]: Israel wird dämonisiert, es wird ein doppelter Maßstab angelegt, und das Land wird als Ganzes delegitimiert“ (https://www.zeit.de/2020/22/felix-klein-holocaust-achille-mbembe-proteste) (14.03.2022). Der Vorwurf wird hier allerdings nicht weiter konkretisiert. Die ebenfalls in dem Interview geäußerte Auffassung Kleins, Mbembe würde Israel sogar als „schlimmer als das südafrikanische Apartheidregime ansehen“, ist durch das oben zitierte Vorwort indes belegt. Hier heißt es im Hinblick auf die israelische Besatzungspolitik: „To be sure, it is not Apartheid, South African Style. It is far more lethal“ (Mbembe 2015, S. viii). Analog Mbembe (2017, S. 85 f.). Zur Analyse einer wenigstens latent antisemitischen Sprachfärbung Mbembes siehe zudem Schwarz-Friesel (2020a).

20 Folgerichtig kritisierte Eva Illouz (2020) sowohl Felix Klein, mit seinem zu tendenziösen Vorwurf an Mbembe dem eigentlichen Kampf gegen den Antisemitismus geschadet zu haben, als auch Mbembe, dass er weder Mitgefühl für die tragische Geschichte der Juden noch ein hinreichendes Gespür für die Komplexität des Israel-Palästina-Konflikts aufbringe. Norbert Reichel (2020) sieht in der Debatte über Mbembes postkolonialen Ansatz zudem eine problematische Opferkonkurrenz bzw. Opferhierarchie am Werk.

21 Zit. nach Hebel (2021). Der Originalkommentar von Thomas Borchert wurde von der* Frankfurter Rundschau *aus der Onlineausgabe entfernt, nachdem Teile der Leserschaft massive Proteste an seiner drastischen Wortwahl („In der Corona-Not mal eben Apartheid zu schlucken, kann man [nur] dem Teufel nachsehen“) geübt hatte.

22 https://www.hrw.org/de/news/2021/01/21/israel-impfstoffe-fuer-palaestinenser-besetzten-gebieten-bereitstellen (14.03.2022). Im April 2021 hat *Human Rights Watch *Israel offiziell der Apartheid angeklagt sowie den ICC angerufen, die systematische Diskriminierung der Palästinenser:innen zu beenden (https://www.theguardian.com/world/2021/apr/27/israel-committing-crime-apartheid-human-rights-watch) (14.03.2022). Auch einige israelische Menschenrechtsorganisationen wie *Yesh Din* oder *B’tselem* kamen im Jahr 2020 zu der Einschätzung, dass die Behandlung der Palästinenser:innen im Westjordanland den Tatbestand der Apartheid erfüllt. Der UN-Ausschuss für die Beseitigung der Rassendiskriminierung (CERD) hat mittlerweile seine Absicht bekundet, den Apartheidvorwurf gegen Israel zu prüfen (https://www.israelnetz.com/gesellschaft-kultur/gesellschaft/2021/05/04/israel-kritisiert-apartheid-untersuchung/) (14.03.2022).

23 Mittlerweile erfolgt die Impfung der Palästinenser im Westjordanland über den Covax-Mechanismus der WHO.

24 Derselbe Ausdruck wird etwa von Neugröschel (2020) und Schwarz-Friesel (2020b) gebraucht, um die aus ihrer Sicht bestehende Unverwechselbarkeit von Antisemitismus und berechtigter Kritik an der israelischen Regierungspolitik auf den Punkt zu bringen.

25 Dass mit dem 3‑D-Test m. E. keine derartige analytische Klarheit zu erzielen ist, wie Süselbeck behauptet, wurde schon am Anfang dieses Abschnitts erläutert.

26 Siehe Anm. 2.

27 Süselbeck selbst kritisiert dazu in seiner Rezension des Bandes von Heilbronn et al. v. a. die darin enthaltenen Aufsätze von Tony Judt und Judith Butler, die die Mitschuld der israelischen Regierung an antisemitischen Ressentiments bzw. die Legitimität von BDS thematisieren.

28 Diesbezüglich sei an dieser Stelle an den Einwand von Leo Strauss gegen die Werturteilsfreiheitslehre Max Webers erinnert, man könne die Handlungen in einem Konzentrationslager nicht ‚sachlich-objektiv‘ beschreiben, ohne sie als „grausam“ zu verurteilen. Selbst nüchtern und ausgewogen gemeinte Stellungnahmen zum Phänomen des Antisemitismus sind mithin immer (auch) normativ aufgeladen zu lesen – der vorliegende Beitrag nicht ausgenommen. Die einschlägigen Debatten sind deshalb stets geeignet, Diskussionspartner ungewollt zu provozieren oder sogar zu verletzen.

29 Siehe Anm. 16.

30 Siehe Anm. 22.

31 Siehe Abschn. 2b.

32 Das israelische Außenministerium konterte die in Anm. 22 zitierten Berichte mit dem Vorwurf, die dahinterstehenden Organisationen würden sich einer *Anti-Israel-Agenda* befleißigen, um Boykotte voranzutreiben und „das Existenzrecht Israels als Nationalstaat des jüdischen Volkes zu untergraben“. (https://www.sueddeutsche.de/politik/israel-palaestinenser-apartheid-human-rights-watch-1.5277547) (14.06.2021).

33 Zur teilweise sehr einseitigen Position von *Human Rights Watch* im Israel-Palästina-Konflikt entgegen den Prinzipien von Universalität und Neutralität siehe z. B. Steinberg (2021).

34 Hier mag man an das Beispiel Ungarn denken, wo die Regierung Orbán zugleich eine antisemitisch imprägnierte Politik gegenüber George Soros führte, wiewohl sie eine enge Partnerschaft mit der Regierung Netanjahu pflegte.

35 Siehe Anm. 28.

36 Siehe Anm. 12.

37 Es ist in diesem Zusammenhang zwar keineswegs ausgeschlossen, dass die eben genannten Formen der ‚Bewusstmachung‘ bis dato unbewusster antisemitischer Ressentiments nicht zwingend zu ihrer Überwindung, sondern eventuell sogar zu deren Verhärtung führen, dennoch sollte evident sein, dass im einen Fall Lernprozesse nicht nur möglich, sondern sogar wahrscheinlich sind, während sie im anderen Fall allenfalls ausnahmsweise vorkommen dürften.

38 Auch hier sei der Vollständigkeit erwähnt, dass das Gleiche wiederum für diejenigen gilt, die bei Kritiker:innen der israelischen Regierungspolitik unbewusst ungerechtfertigte Antisemitismusvorwürfe erheben. Das subjektive Anliegen, wirklich nur antisemitische Vorurteile aufdecken und bekämpfen zu wollen, ist in diesem Zusammenhang unbedingt ernst zu nehmen. Gleichwohl ist ggf. darauf aufmerksam zu machen und zu begründen, wann eine subjektive Argumentation intersubjektiv dazu angetan ist, die israelische Regierung gegenüber Kritik zu immunisieren, nicht zuletzt, um zu vermeiden, dass sich jemand zum Rechtfertiger einer Politik aufschwingt, der dies im Grunde genommen gar nicht will.

39 Zit. nach https://www.spiegel.de/politik/naehrboden-fuer-antisemitismus-a-28143c49-0002-0001-0000-000022078169 (17.03.2021).

40 Siehe dazu das Interview Blüms im *Stern *vom 20.06.2002, in dem er vom „Knüppel“ des Antisemitismusvorwurfs sprach.

41 Siehe dazu z. B. Waldmann (1998), Jacobson (2006) sowie Art und Richardson (2007). Durch eine solche Provokation von Überreaktionen versuchen militärisch unterlegene Terrorist:innen für gewöhnlich, sich selbst als Opfer darzustellen und den von ihnen gewaltsam attackierten Staat als illegitimen Angreifer erscheinen zu lassen.

42 Dazu nochmals die Anm. 37: Eine subjektiv unbewusste, dem Grund nach *ungewollte* antisemitische Perspektive impliziert wenigstens die logische Option, im Rahmen einer versachlichten Debatte argumentativ entkräftet werden zu können und somit Lernprozesse loszutreten, die bei bewussten, manifesten und subjektiv als legitim eingestuften Ressentiments nahezu ausgeschlossen scheinen.

43 Zum Alleinstellungsmerkmal der Demokratie, aufgrund der in ihr periodisch changierenden Macht Fehler ggf. wiedergutzumachen und v. a. krasse Fehlentwicklungen zu bereinigen, siehe bereits die klassische Analyse von Alexis de Tocqueville (1976, S. 259 f.). Zu jenem ‚mäßigenden‘ Grundcharakterzug der Demokratie im Gegensatz zu autoritären oder totalitären Regimen auch Schmidt (2010, S. 117 f.).

44 Dazu nach wie vor das sog. *Wollheim-Paradox* (Wollheim 1962).

45 Siehe dazu vor allem den Essay *Vom Wesen und Wert der Demokratie* in der maßgeblichen Ausgabe von 1929 (Kelsen 2006).

46 Diesbezüglich hat besonders Jacques Rancière (1996, S. 101, 2002) vollkommen zu Recht betont, dass jede demokratische Entscheidung im ‚Namen des Volkes‘ streng genommen einen usurpatorischen Akt beinhaltet, da kein demokratischer Akteur oder Organ, keine Gruppe oder Institution tatsächlich für sich beanspruchen kann, ‚den‘ Willen des Volkes zu repräsentieren, nicht einmal die in plurale Identitäten aufgespaltene Bevölkerung selbst. Streit und Unvernehmen bilden daher eine Art aporetische Substanz der Demokratie, deren Einheit – wie schon Claude Lefort (1990) unterstrich – allenfalls auf der symbolisch-ästhetischen Ebene stattfinden kann.

47 Siehe dazu u. a. die Diagnosen von Goodhart (2017), de Wilde et al. (2019) und Klein (2020).

48 So beklagte etwa die (säkulare) Politikerin Ajelet Schaked, zur Zeit der Verabschiedung des Nationalstaatsgesetzes Abgeordnete der nationalreligiösen Partei *HaBajit haJehudi* (Jüdisches Heim) und trotz ihres Austritts im Dezember 2018 bis 2019 Justizministerin im Kabinett Netanjahu, dass die ‚konstitutionelle Revolution‘ Israel seines jüdischen Charakters beraubt habe. Sie forderte daher explizit: „Wir müssen den jüdischen Charakter des Staates schützen, auch wenn das bedeutet, Menschenrechte zu opfern“ (zit. nach Lintl 2018). Dazu auch https://www.i24news.tv/en/news/israel/167536-180212-israeli-minister-says-israel-to-stay-jewish-even-at-expenses-of-human-rights (16.03.2022).

49 Hierzu ausführlich Hidalgo (2014, Kap. 3.4).

50 Zu dieser Motivation siehe die Stellungnahme von Yariv Levin (Likud), einem der Architekten des Nationalstaatsgesetzes (https://www.commentarymagazine.com/articles/harkov-lahav/making-the-jewish-state-a-jewish-state/) (16.03.2022).

51 Zur heute in vielen Demokratien zu beobachtenden problematischen Nähe zwischen politisch illiberalen und radikalen religiösen Kräften siehe v. a. den Band von Hennig und Weiberg-Salzmann (2021).

52 Zit. nach Holmes (2020).

53 Hier ließen sich durchaus Parallelen zur späteren These von Max Horkheimer und Theodor Adorno in der *Dialektik der Aufklärung* (1944) aufzeigen, wonach der Antisemitismus nichts anderes als eine negative, barbarische, unüberwindbare Begleiterscheinung der in der modernen Gesellschaft ausgeprägten Denkform bedeute.

54 Dan Diner (2019, S. 460) schlägt deswegen vor, heute besser zwischen einem historisch verdichteten Antisemitismus und dem eher amorphen, „antisemitisierenden“ Charakter gegenwärtiger Ressentiments gegenüber Jüdinnen und Juden eine Trennlinie zu ziehen. Die von Diner indes zugleich festgestellten „Risse im Sarkophag“ des tabuisierten Antisemitismus, die nicht zuletzt im Kontext des Israel-Palästina-Konflikts sichtbar werden, sind umgekehrt aber wiederum als Argument *für* die Relevanz einer Unterscheidung von manifesten (bewussten) und latenten (unbewussten) Vorurteilen gegenüber Jüdinnen und Juden zu lesen. Nochmals auf einem anderen, hier nicht zu verhandelnden Blatt steht hingegen Diners Analyse der heute zunehmenden, paradoxen „Gegenläufigkeit von israelischer [zionistischer] Staatsräson und diasporischer jüdischer Existenz“, wobei letztere „ihren Halt in den [überstaatlichen] Bürger- und Menschenrechte“ finde, während erstere sich nicht nur kaum „an überhandnehmenden judenfeindlichen Stimmungen“ störe, sondern bisweilen sogar mit „ausgemachten Antisemiten“ gemeinsame Sache mache (Diner 2019, S. 488). Dazu auch Anm. 34.
